# Mississippi State Medical Association

**Published:** 1898-04

**Authors:** J. R. Tackett

**Affiliations:** Secretary; Biloxi, Miss.


### Mississippi State Medical Association.

BILOXI, MISS., March 14, 1898.

DEAR DOCTOR:—The thirty-first annual meeting of the Mississippi State Medical Association will convene in Jackson, Wednesday, April 20th, 1898.

You are urged lo be present and contribute to the success of the meeting. Please forward me the title of the paper you intend reading before the Association, by the first of April, that it may appear on the programme.

Yours truly,

J. R. TACKETT, Secretary.

